# Mechanisms of resistance to KRASG12C inhibitors in KRASG12C-mutated non-small cell lung cancer

**DOI:** 10.3389/fonc.2024.1328728

**Published:** 2024-09-05

**Authors:** Ali Chour, Anne-Claire Toffart, Elodie Berton, Michael Duruisseaux

**Affiliations:** ^1^ Respiratory Department and Early Phase (EPSILYON), Louis Pradel Hospital, Hospices Civils de Lyon Cancer Institute, Lyon, France; ^2^ Oncopharmacology Laboratory, Cancer Research Center of Lyon, UMR INSERM 1052 CNRS 5286, Lyon, France; ^3^ Université Claude Bernard, Université de Lyon, Lyon, France; ^4^ Service de Pneumologie et Physiologie, Centre Hospitalier Universitaire Grenoble Alpes, Grenoble, France; ^5^ Institute for Advanced Biosciences, UGA/INSERM U1209/CNRS 5309, Université Grenoble Alpes, Grenoble, France

**Keywords:** non-small cell lung cancer, KRASG12C mutation, KRASG12C inhibitor resistance, translational research, sotorasib, adagrasib

## Abstract

The KRAS protein, a product of the KRAS gene (V-ki-ras2 Kirsten rat sarcoma viral oncogene homolog), functions as a small GTPase that alternates between an active GTP-bound state (KRAS(ON)) and an inactive GDP-bound state (KRAS(OFF)). The *KRAS^G12C^* mutation results in the accumulation of KRASG12C(OFF), promoting cell cycle survival and proliferation primarily through the canonical MAPK and PI3K pathways. The *KRAS^G12C^* mutation is found in 13% of lung adenocarcinomas. Previously considered undruggable, sotorasib and adagrasib are the first available OFF-state KRASG12C inhibitors, but treatment resistance is frequent. In this review, after briefly summarizing the KRAS pathway and the mechanism of action of OFF-state KRASG12C inhibitors, we discuss primary and acquired resistance mechanisms. Acquired resistance is the most frequent, with "on-target" mechanisms such as a new *KRAS* mutation preventing inhibitor binding; and "off-target" mechanisms leading to bypass of *KRAS* through gain-of-function mutations in other oncogenes such as *NRAS*, *BRAF*, and *RET*; or loss-of-function mutations in tumor suppressor genes such as *PTEN*. Other "off-target" mechanisms described include epithelial-to-mesenchymal transition and histological transformation. Multiple co-existing mechanisms can be found in patients, but few cases have been published. We highlight the lack of data on non-genomic resistance and the need for comprehensive clinical studies exploring histological, genomic, and non-genomic changes at resistance. This knowledge could help foster new treatment initiatives in this challenging context.

## 1 Introduction

The RAS (rat sarcoma viral oncogene) protein is a small guanosine triphosphate hydrolase (GTPase) that alternates between an active GTP-bound state (RAS(ON)) and an inactive GDP-bound state (RAS(OFF)). Guanine nucleotide exchange factors (GEFs) and GTPase-activating proteins (GAPs) regulate the transition from RAS(ON) to RAS(OFF) and from RAS(OFF) to RAS(ON) (1). RAS(ON) promotes several important signaling pathways, primarily the RAS-RAF-MEK-ERK pathway and the RAS-PI3K-AKT-mTOR pathway, thus playing an important role in cell survival and cell cycle proliferation (2).

Growth factors can induce rapid dimerization and autophosphorylation of their receptors (GFRs). Specific tyrosine residues in noncatalytic regions of autophosphorylated GFRs can interact with the SH2 domain of the Grb2 protein. Coupled with the Son of Sevenless (Sos) protein, the Grb2-Sos complex stimulates the exchange of GDP for GTP on RAS, thus leading to RAS(ON) promotion. The Grb2-Sos complex is the primary GEF (3, 4).

Even though RAS possesses intrinsic low GTPase activity, additional proteins are needed to accelerate GTP hydrolysis. Those GAPs (such as RASA1, neurofibromin, or DAB2IP) aid, via their arginine finger, in the structural rearrangement and assembly of a catalytically competent active site, leading to nucleotide release (4, 5). Regulation of RAS signaling thus depends on a balance between GEFs and GAPs.

The RAS(ON) protein binds to RAS binding domains (RBD) located on RAS effectors, which are proteins with a strong affinity to RAS(ON). RAF (rapidly accelerated fibrosarcoma) is a critical RAS effector that triggers the RAS-RAF-MEK-ERK pathway. PI3K is another important RAS effector that activates the RAS-PI3K-AKT-mTOR pathway (4).

Four isoforms of the RAS protein are found in humans: HRAS, NRAS, KRAS4A, and KRAS4B (6), with RAS mutations detected in 19% of cancer patients (75% in *KRAS*, 17% in *NRAS*, and 7% in *HRAS*) (7).

*KRAS* mutations are frequent in lung adenocarcinomas, accounting for 43% of cases (8), while *NRAS* and *HRAS* mutations account collectively for approximately 1.2% of cases. Furthermore, 80% of NSCLC-*KRAS* mutations involve a glycine on position 12 substitution (*KRAS^G12C^*, *KRAS^G12V^*, *KRAS^G12D^* …) and 11% involve a glycine on position 13 substitution (*KRAS^G13C^*, *KRAS^G13D^*, *KRAS^G13R^* …).

The most frequent *KRAS* mutation is *KRAS^G12C^*, present in 13% of lung adenocarcinomas (9). This glycine at position 12 substitution to cysteine in the KRASG12C protein prevents interaction with GAPs through steric blockade, resulting in reduced GTPase activity responsible for the accumulation of active KRASG12C-GTP bound protein (KRASG12C(ON)). Unlike other oncogenic mutations such as *EGFR*'s classical L858R exon 21 mutation or exon 19 deletions, *KRAS* mutations are predominantly seen in the majority of patients with a history of smoking and co-mutations are not rare (mainly *TP53*, *STK11*, and *KEAP1*). However, *KRAS* mutations are considered mutually exclusive with other NSCLC driver alterations, such as *EGFR* mutations, *EML4*-*ALK* fusions, or *ROS1* fusions.

**Figure 1:**
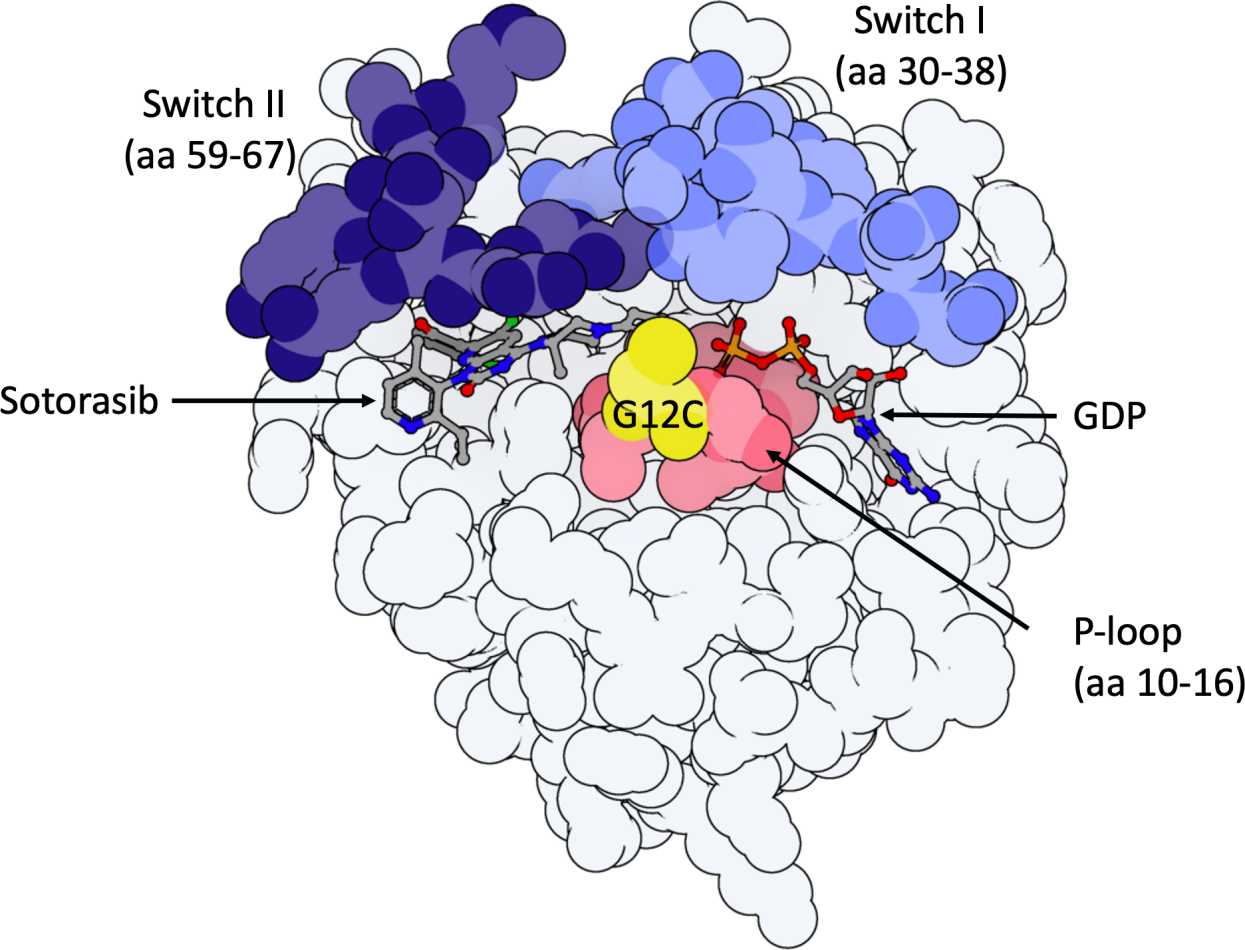
Model of KRASG12C-protein in the inactive GDP-bound state with important domains highlighted. 3D molecule was rendered with ProteinImager (http://3dproteinimaging.com) based on the crystal structure (PBD ID: 6OIM, rcsb.org/structure/6OIM). The structure of the *KRAS* gene comprises a G-domain coding region and a hypervariable region, including the conserved CAAX motif, a membrane anchor sequence (C: cysteine, A: aliphatic amino acids, X: any amino acid and residues coding for a lipid tail; not shown here). Selected structural regions of the KRAS protein G-domain are highlighted: the phosphate-binding loop (P-loop, amino acids (aa) 10 to 16) and the two switch regions (switch I (aa 30 to 38) and switch II (aa 59 to 67)). Both switch regions change conformation to make hydrogen bonds with the gamma-phosphate in GTP-bound-KRAS. Sotorasib is observed in its binding pocket (P2, behind Switch II region, near mutated Cysteine-12). The mutated Cystein-12 residue is shown in yellow. Adagrasib interacts with the P2 binding pocket in the same way as sotorasib.

Long deemed undruggable due to its lack of apparent hydrophobic pockets and its picomolar affinity for GTP/GDP, new KRASG12C inhibitors are finally under investigation in preclinical and clinical studies (10). These treatments bind covalently to an H95 residue located in an allosteric binding pocket behind switch-II, referred to as P2, near the mutated cysteine 12, in the inactive KRASG12C-GDP bound protein (KRASG12C(OFF) (**Figure 1**). This covalent binding in the P2 pocket induces the blocking of nucleotide exchange from GDP to GTP (10) thereby inhibiting RAS-effector interaction in *KRAS^G12C^* mutant cells. Despite the *KRAS^G12C^* mutation inducing the accumulation of KRASG12C(ON) protein, 25% of proteins in each cell remain in a GDP-bound inactive state, explaining the potential for protein inhibition (11).

Sotorasib is the first-in-class OFF-state KRASG12C inhibitor, available in France since early 2021 through an early access program. The phase III open-label randomized controlled trial (RCT) CodeBreak 200 demonstrated superior progression-free survival (PFS) over docetaxel (median PFS 5.6 months (4.3-7.8) in the sotorasib group vs 4.5 months (3.0-5.7) in the docetaxel group; 12-month PFS rate of 24.8% with sotorasib vs 10.1% with docetaxel) (12). However, the overall survival (OS) did not reach statistical significance, partly due to a decrease in sample size after protocol amendment and a 26% cross-over rate in the docetaxel group.

Adagrasib is another OFF-state KRASG12C inhibitor with results from the recently published phase I/II KRYSTAL-1 study (13) showing promising outcomes with adagrasib in the same second-line setting as sotorasib. A phase III randomized controlled trial (NCT04685135) is in progress. Additionally, studies investigating sotorasib and adagrasib (alone or combined with chemo- or immunotherapy) in the first-line and the second-line settings are ongoing (34 trials listed on clinicaltrials.gov). A summary of the main efficacy results in the second-line setting is described in **Table 1**.

**Table 1:** Efficacy data from Phase II/III trials involving KRASG12C inhibitors sotorasib and adagrasib in pretreated advanced non-small cell lung cancer.

KRASG12C Inhibitor	Study	Phase	Control	Number of patients		Objective Response^a^ - %, (95% CI)		Median duration of response^b^, mo (95% IC)		Median PFS, mo (95% IC)		Median OS, mo (95% IC)	
				Control	KRASG12Ci	Control	KRASG12Ci	Control	KRASG12Ci	Control	KRASG12Ci	Control	KRASG12Ci
**Sotorasib**	CodeBreak 100	II^15^			126		37.1 (28.6-46.2)		11.1 (6.9-NE)		6.8 (5.1-8.2)		12.5 (10-NE)
	CodeBreak 200	III^13^	Docetaxel	174	174	13.2 (8.6-19.2)	28.1 (21.5-35.4)	6.8 (4.3-8.3)	8.6 (7.1-18.0)	4.5 (3.0-5.7)	5.6 (4.3-7.8)	11.3 (9.0-14.9)	10.6 (8.9-14.0)
**Adagrasib**	Krystal-1	II^14^			116		42.9 (33.5-52.6)		8.5 (6.2-13.8)		6.5 (4.7-8.4)		12.6 (9.2-19.2)

a Objective response was defined as a complete or partial response.

b Duration of response was evaluated based on patients with a complete or partial response. KRASG12Ci, KRASG12C inhibitor; CI, confidence interval; mo, months; NE, not evaluated.

Despite promising efficacy, resistance to OFF-state KRASG12C inhibitors occurs in virtually all patients. Notably, one-third of patients experienced early disease progression (PFS < 3 months) on sotorasib in the CodeBreaK100 study. Primary and early adaptative resistance mechanisms may drive early disease progression (14). Recent preclinical and clinical datasets suggest that resistance mechanisms to the KRASG12C inhibitors sotorasib and adagrasib may be categorized into two distinct groups: genetic and non-genetic mechanisms, which can explain early and delayed resistance at different levels.

This review aims to describe and examine emerging mechanisms of resistance to OFF-state KRASG12C inhibitors in *KRAS^G12C^*-mutated non-small cell lung cancer (NSCLC) and to demonstrate how this body of data is shaping the therapeutical development in KRASG12C targeting.

## 2 Genetic mechanisms of resistance to OFF-state KRASG12C inhibitors

### 2.1 Genetic determinants of primary resistance

Genomic alterations were correlated with long-term benefit (PFS ≥ 12 months) versus early progression (PFS < 3 months) in the CodeBreaK100 study dataset (14). The most significant enrichment in patients with early progression was observed with mutant *KEAP1*. Aggregated with other emerging data, co-occurring mutations in *KEAP1, SMARCA4*, and *CDKN2A* are associated with worse clinical outcomes with sotorasib or adagrasib therapy (14–16). The biological mechanisms driving early progression in this subgroup of patients with co-occurring *KEAP1*, *SMARCA4*, and *CDKN2A* mutations are not clearly understood.

### 2.2 Genetic mechanisms of acquired resistance

**Figure 2:**
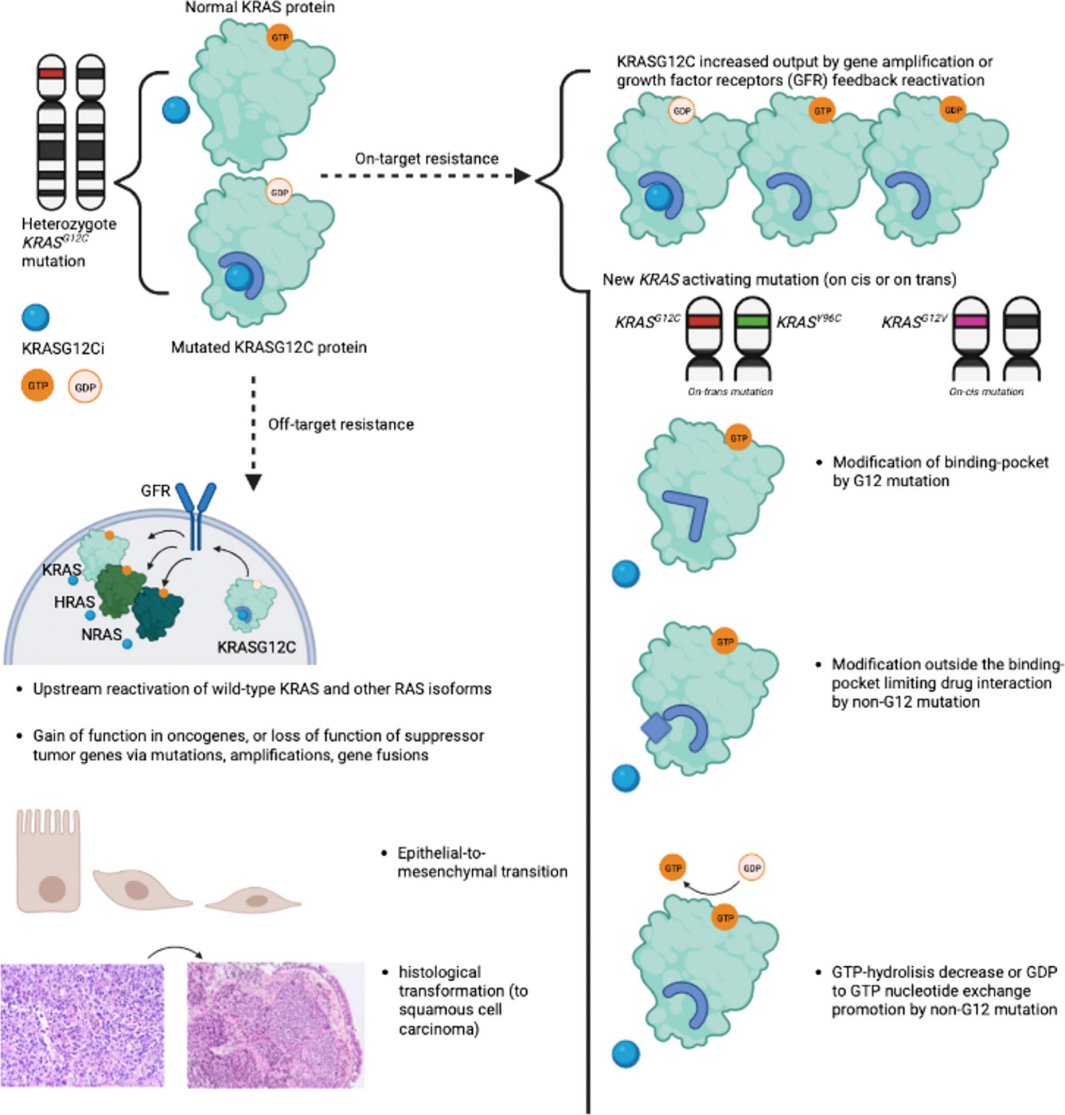
Summary of "on-target" and "off-target" resistance mechanisms to OFF-state KRASG12C inhibitors. Summary of "on-target" and "off-target" resistance mechanisms described with OFF-state KRASG12C inhibitors sotorasib and adagrasib. "On-target" resistance encompasses new *KRAS* activating mutations and increased KRASG12C output due to growth factor receptors' feedback reactivation. "Off-target" resistance mechanisms include amplification or mutations of other oncogenes, upstream reactivation of wild-type KRAS and other RAS isoforms, epithelial-to-mesenchymal transition, and adeno-to-squamous transition. The illustration was created with BioRender.com. KRASG12Ci, OFF state KRASG12C inhibitor; GFR, Growth Factor Receptor.

Mechanisms of acquired resistance have been partly described following treatment with sotorasib and adagrasib. Resistance has been categorized as "on-target" or "off-target", with the majority of published data focusing on "on-target" mechanisms. **Figure 2** summarizes acquired mechanisms of resistance to OFF-state KRASG12C inhibitors (11).

#### 2.2.1 "On-target" genetic mechanisms of resistance to OFF-state KRASG12C inhibitors

"On-target" genetic mechanisms of resistance to OFF-state KRASG12C inhibitors encompass mutations of the *KRAS* ^G12C^ codon to another mutant variant on the same allele (*cis*) or a secondary KRAS mutation on the *trans*-*KRAS* allele. CfDNA analysis of a 67-year-old patient with *KRAS^G12C^*-mutant NSCLC after progression on adagrasib showed a new *(trans) KRAS^G12V^* mutation (11), coexisting with the *(cis) KRAS^G12C^* mutation and probably arising from the wild-type *KRAS* allele. The persistence of a wild-type *KRAS* allele in multiple *KRAS*-mutated lung cancer cell lines was observed in preclinical studies (17). Acquired *KRAS^G12D/R/V/W^* mutations in other patients led to the reactivation of the KRAS downstream pathway (18). Non-*KRAS^G12^* mutations affecting switch II pocket and precluding drug binding, such as *KRAS^Y96C^*, *KRAS^R68S^*, or *KRAS^H95D/Q/R^*, were described (18). Other KRAS mutations, like *KRAS^G13D^* or *KRAS^A59S^*, induce resistance by decreasing GTP hydrolysis or promoting GDP to GTP nucleotide exchange (18).

*KRAS^G12C^* allele amplification or copy number gain were the only identifiable resistance mechanisms in two patients treated with adagrasib (18).

Upstream reactivation of associated proteins such as Aurora Kinase A (AURKA), a serine/threonine kinase essentially involved in mitosis and DNA repair, has been shown to facilitate effector activation by stabilizing the interaction between newly formed KRASG12C protein and RAF, thus aiding cell cycle progression in *in vitro* models (19).

In summary, the inhibition of KRASG12C(OFF) downregulates physiological negative feedback mechanisms, leading to the upregulation of GFR (19). Similar resistance mechanisms have already been described with MEK inhibitors (20) and BRAF inhibitors (21).

#### 2.2.2 "Off-target" genetic mechanisms of resistance to OFF-state KRASG12C inhibitors

"Off-target" genetic mechanisms of resistance to OFF-state KRASF12C inhibitors include:

- Amplifications or mutations of upstream RTK genes (such as *EGFR*).
- Bypass of KRASG12C through activating mutation in downstream pathway components, including *MEK*, *BRAF*, or *PI3KCA*.
- Activating mutation in *NRAS* or *HRAS*.

In an analysis of 38 patients with *KRAS^G12C^-*mutant cancers resistant to adagrasib (18), a putative resistance mechanism was seen in only 17 patients. These mechanisms included multiple acquired bypass resistances (such as *EGFR*, *RET*, *NRAS*, *BRAF*, *PI3K*, and *PTEN* mutations), acquired gene fusions (e.g., *EML4-ALK*), or amplification (e.g., *MET*). An important limitation of this study was the limited number of tissue samples, with most analyses conducted using cfDNA sequencing. Interestingly, multiple co-resistance mechanisms were found in patients.

For example, one patient developed a *KRAS^G12D^* and *KRAS^Q61H^* mutation (an "on-target" resistance mechanism) associated with a *BRAF^V600E^* bypass mutation and a *CCD6-RET* fusion (an "off-target" resistance mechanism). The MET amplification was found *in vitro* to bypass the RAS pathway through the HGF/MET pathway, leading to AKT and ERK activation, but also reactivating the RAS-BRAF-MEK-ERK pathway through other RAS isoforms (22)

## 3 Non-genetic mechanisms of resistance to OFF-state KRASG12C inhibitors

A large majority of patients have no identifiable genetic mechanisms of resistance to OFF-state KRASG12C inhibitors, suggesting that resistance may arise from non-genetic alterations.

### 3.1 Upstream reactivation of growth factor receptors

Rapid adaptative resistance may be driven by GFR reactivation (19, 23) The upstream reactivation of GFRs (including multiple tyrosine kinase receptors (RTKs)) not only increases KRASG12C output but also activates wild-type KRAS and other RAS isotypes (NRAS and HRAS), which can at least partially restore MAPK signaling. This rebound mechanism, with higher concentrations of NRAS(ON) and HRAS(ON) and downstream pathway reactivation (shown by phosphorylation of downstream MAPK proteins ERK (extracellular signal-regulated kinases) and RSK (ribosomal s6 kinase)), was seen in the first 48h following KRASG12C inhibition in *KRAS^G12C^*-mutant cell lines (19). RTKs such as EGFR, but also HER2, FGFR, and cMET were activated with different levels in different cell lines. RTK reactivation can activate the downstream ERK pathway via SHP2 tyrosine phosphatase interaction with Grb2. This has been shown *in vitro* with the rapid increase of SHP2 activation in multiple *KRAS*-mutant lung, colon, and pancreatic cell lines after initial reduction following MEK inhibition (23).

### 3.2 Histological transdifferentiation and cell lineage plasticity

Histological transdifferentiation and cell lineage plasticity may play a role in the resistance to OFF-state KRASG12C inhibitors. Two *KRAS^G12C^* mutant lung adenocarcinomas treated with adagrasib showed squamous cell carcinoma histology in biopsies at progression, with no genomic alterations explaining the resistance otherwise (18). Transcriptomic and genomic analysis on pre-treatment biopsies from patients in the KRYSTAL-1 trial revealed that patients presenting a baseline high expression of squamous cell carcinoma-related genes and *STK11/LKB1* co-mutations had a shorter treatment duration with adagrasib (24). *STK11/LKB1* co-mutations are frequent in *KRAS^G12C^* mutant lung adenocarcinomas, and *LKB1* is a regulator of chromatin accessibility linked with cellular plasticity (25, 26). Its inactivation induced squamous transition in *KRAS^G12D^*-mutant lung adenocarcinoma cell lines. In a preclinical study, adagrasib-resistant *KRAS/LKB1* mutant NSCLC showed enrichment in adenosquamous transition-associated genes, including *Wnt4, Sfn, Aqp3*, and *Krt6a* (24). In another preclinical study, KRAS inhibition was associated with the transition of lung adenocarcinoma alveolar type 2 cells to alveolar type 1 (AT1) cells. AT1 cells exhibited less dependency on *KRAS* and more quiescent activity (27). Both mechanisms have been described with EGFR and ALK inhibitors in *EGFR*-mutation-positive and *ALK*-fusion-positive NSCLC models (28).

### 3.3 Epithelial-to-mesenchymal transition

Epithelial-to-mesenchymal transition (EMT) has been observed in KRASG12C-mutated cancer cell lines after induced resistance to sotorasib. EMT, the process by which epithelial cells acquire mesenchymal features, is associated in cancer with tumor invasion, initiation, metastasis, and resistance to therapy (29), notably with EGFR and ALK inhibitors in *EGFR*-mutation-positive and *ALK*-fusion-positive NSCLC models (30, 31). EMT can be induced by numerous biological drivers such as TGFβ, TNF-α, HIF-α, Wnt signaling, interleukins, Hedgehog, and Hippo pathways (32). Genes related to EMT were enriched in sotorasib-resistant NSCLC cell lines (33). Induction of EMT via chronic TGFβ treatment was sufficient to induce resistance to sotorasib. In this population of EMT-induced cells, rebound activation of ERK and S6 was observed. Cell growth was inhibited after the addition of a PI3K inhibitor to sotorasib, implying that KRASG12C-independent AKT activation is a cause of resistance to sotorasib in EMT-induced *KRAS^G12C^*-mutant NSCLC cell lines. These results were confirmed in a xenograft mouse model. EMT dependence on CDK-4 (cyclin-dependent kinase 4) has also been described in *in vitro* models, with promising efficacy for CDK4 inhibitors in reducing tumor volume in a murine model with autochthonous mesenchymal-like lung cancer with a *KRAS^G12D^* mutation (34).

### 3.4 Tumor microenvironment

The tumor microenvironment (TME) associated with *KRAS*-mutant tumor cells is highly immunosuppressive (35). The TME is transiently converted to a less immunosuppressive state following RAS inhibition and may increase susceptibility to immunotherapies.

Due to a lack of data, other non-genomic resistance mechanisms to KRASG12C cannot be described to date. For example, no data about epigenetic dysregulation in this context are yet available.

## 4 Lessons from known mechanisms of resistance to OFF-state KRASG12C inhibitors and therapeutical perspectives

Co-mutations in key tumor suppressor genes (*KEAP1*, *SMARCA4*, and *CDKN2A*) define different subgroups of KRASG12C-mutant NSCLC with clearly different clinical outcomes with OFF-state KRASG12C inhibitors. These co-mutations may serve as biomarkers of clinical activity for OFF-state KRASG12C inhibitors, should be integrated as stratification factors in clinical trials, and may guide escalated or de-escalated treatment strategies. The key role of co-mutations in defining patient subgroups with primary resistance and the diversity of on-target mechanisms of resistance explaining early and acquired resistance to OFF-state KRASG12C inhibitors suggest that KRASG12C-mutant lung adenocarcinomas are highly genetically heterogeneous. This intra-tumoral genetic heterogeneity could partially explain the high proportion of early progressors and the low proportion of durable responders. Pan-RAS/KRAS inhibitors may prevent the emergence of acquired on-target mutations on *KRAS* and partly address the role of genetic heterogeneity in resistance to KRAS inhibition. For example, RMC-6236 is an ON-state RAS multi-selective noncovalent inhibitor of the active, GTP-bound state of both mutant and wild-type variants of RAS isoforms (36). RMC-6236 exhibited potent anticancer activity across RAS-addicted tumor models and showed early clinical activity (36, 37).

Amplifications or mutations of upstream RTK and upstream reactivation of GFR that drive RTK-driven pathway rebound can be prevented by RMC-6236 and ON/OFF-state direct inhibitors such as FMC-376 (38).

Upstream and downstream dysregulation of the RAS signaling pathway induced by OFF-state KRASG12C inhibitors offer attractive targets for combination therapies. SOS1 and SHP2 are activated by RTK and regulate the switch of RAS from the OFF state to the ON state. Inhibition of SOS1 and SHP2 activity stabilizes GDP-bound RAS in an inactive form (39–41). Several combinations including SOS1 and SHP2 inhibitors are under clinical evaluation (42).

Other combinations under clinical evaluation include anti-PD-(L)1 with sotorasib or adagrasib (43). Sotorasib induced a proinflammatory tumor microenvironment highly sensitive to immunotherapy in a preclinical study (44). The combination of sotorasib and anti-PD-1 therapy resulted in a higher response rate and more durable responses in mice compared to sotorasib monotherapy or anti-PD-1 monotherapy (44). However, this strategy with sotorasib is limited in clinical practice due to higher rates of side effects, mainly hepatotoxicity, when sotorasib is prescribed in combination with or following anti-PD(L)1 therapy (45, 46). Preliminary results from the KRYSTAL-7 phase II trial did not show a higher rate of hepatotoxicity with adagrasib and pembrolizumab, hinting at a possible non-class effect (47). Multiple ongoing studies are investigating OFF-state KRASG12C inhibitors and anti-PD(L)1 agents (43).

## 5 Conclusion

Several phase III trials comparing OFF-state KRASG12C inhibitors (sotorasib, adagrasib, divarasib, olomorasib, opnurasib) to standard-of-care chemotherapy and immunotherapy in NSCLC are ongoing (47–49). They will provide highly valuable data on the benefit of these drugs, the optimal sequencing strategy, and hopefully, insights into mechanisms of resistance to these drugs. The design of these trials mainly relies on patient selection according to *KRAS^G12C^* mutation and PD-L1 biomarkers.

The understanding of resistance mechanisms to KRASG12C inhibitors is in its early stages. It relies on data generated with OFF-state KRASG12C inhibitors, mainly sotorasib and adagrasib, but provides an essential framework for future rationally designed therapeutic development. Thus, the early emergence of RAS-MAPK signaling reactivation through acquired resistance mutations and upstream reactivation of GFR underscores the strong need for RAS signaling in *KRAS^G12C^*-mutant cancers as well as the major role of tumor heterogeneity in resistance to OFF-state KRASG12C inhibitors. As a result, therapeutic strategies based on strong inhibition of RAS signaling (ON- or ON/OFF-state RAS inhibitors), broad inhibition of the RAS pathway (pan-RAS inhibitors), and combination strategies that target upstream or downstream of the RAS pathway are relevant and currently being evaluated in clinical trials. There is a strong biological rationale supporting KRASG12C inhibitors and immunotherapy, specifically anti-PD-(L1) agents, and combination strategies, and multiple clinical trials are evaluating the safety and clinical activity of such combinations. Overall, the clinical evaluation of drug combination strategies is the way to address the problem of primary and acquired resistance to KRASG12C inhibitors. These efforts should focus on understanding the biology driving KRASG12C-targeting clinical efficacy and selecting the most effective and relevant combination strategy and predictive biomarker of efficacy for future development, especially through phase III trials. Therapeutic platforms such as Master Protocols can effectively evaluate multiple combination strategies in KRAS^G12C^-mutant NSCLC. ctDNA sample analysis can be a highly valuable tool for identifying early signals of efficacy and understanding mechanisms of resistance that may drive future preclinical and clinical development.
